# Factors influencing microbial colonies in the air of operating rooms

**DOI:** 10.1186/s12879-017-2928-1

**Published:** 2018-01-02

**Authors:** Ling Fu Shaw, Ian Horng Chen, Chii Shya Chen, Hui Hsin Wu, Li Shing Lai, Yin Yin Chen, Fu Der Wang

**Affiliations:** 1 Department of Nursing, Taipei Veterans General Hospital, Taipei, Taiwan; 2 College of Nursing, National Yang-Ming University, Taipei, Taiwan; 3 Taipei City Hospital, Taipei, Taiwan; 4 Taipei City Zhongzheng District Health Center, Taipei, Taiwan; 5 Department of Family Medicine, Puli Christian Hospital, Nan-Tou, Taiwan; 6 Department of Infection Control, Taipei Veterans General Hospital, No. 201, Section 2, Shih–Pai Road, Taipei, 11217 Taiwan; 7 Division of Infectious Diseases, Department of Medicine, Taipei Veterans General Hospital, Taipei, Taiwan; 8 School of Medicine, National Yang–Ming University, Taipei, Taiwan

**Keywords:** Microbial colonies, Air quality, Operating room

## Abstract

**Background:**

The operating room (OR) of the hospital is a special unit that requires a relatively clean environment. The microbial concentration of an indoor OR extrinsically influences surgical site infection rates. The aim of this study was to use active sampling methods to assess microbial colony counts in working ORs and to determine the factors affecting air contamination in a tertiary referral medical center**.**

**Methods:**

This study was conducted in 28 operating rooms located in a 3000-bed medical center in northern Taiwan. The microbiologic air counts were measured using an impactor air sampler from May to August 2015. Information about the procedure-related operative characteristics and surgical environment (environmental- and personnel-related factors) characteristics was collected.

**Results:**

A total of 250 air samples were collected during surgical procedures. The overall mean number of bacterial colonies in the ORs was 78 ± 47 cfu/m^3^. The mean number of colonies was the highest for transplant surgery (123 ± 60 cfu/m^3^), followed by pediatric surgery (115 ± 30.3 cfu/m^3^). A total of 25 samples (10%) contained pathogens; *Coagulase-negative staphylococcus* (*n* = 12, 4.8%) was the most common pathogen. After controlling for potentially confounding factors by a multiple regression analysis, the surgical stage had the significantly highest correlation with bacterial counts (*r* = 0.346, *p* < 0.001). Otherwise, independent factors influencing bacterial counts were the type of surgery (29.85 cfu/m^3^, 95% CI 1.28–58.42, *p* = 0.041), site of procedure (20.19 cfu/m^3^, 95% CI 8.24–32.14, *p* = 0.001), number of indoor staff (4.93 cfu/m^3^, 95% CI 1.47–8.38, *p* = 0.005), surgical staging (36.5 cfu/m^3^, 95% CI 24.76–48.25, *p* < 0.001), and indoor air temperature (9.4 cfu/m^3^, 95% CI 1.61–17.18, *p* = 0.018).

**Conclusions:**

Under the well-controlled ventilation system, the mean microbial colony counts obtained by active sampling in different working ORs were low. The number of personnel and their activities critically influence the microbe concentration in the air of the OR. We suggest that ORs doing complex surgeries with more surgical personnel present should increase the frequency of air exchanges. A well-controlled ventilation system and infection control procedures related to environmental and surgical procedures are of paramount importance for reducing microbial colonies in the air.

## Background

The operating room (OR) of the hospital is a special unit that requires a relatively clean environment, with the least number of microorganisms in the air [1]. Ambient air may be contaminated with or carry significant levels of a variety of potentially detrimental microorganisms [2, 3]. Exogenous flora are primarily aerobes, especially gram-positive organisms (e.g., *staphylococci* and *streptococci*) [4, 5]. The type and quantity of microorganisms in the air are some of the factors that cause infection [6]. Early reports estimated that airborne transmission accounted for 10%–24% of surgical site infections (SSI) [3]. Most modern hospitals following the guidelines for the prevention of SSIs recommend that air flow conditions in hospital ORs are controlled and regulated to remove airborne microorganisms to help control infections [5, 7–9]. Nonetheless, some reports indicated that microbial contamination concentrations exceed the recommended standard values [10, 11]. These also increase the risk of infection, leading to medical consumption [12–14].

In addition to environmental factors, personnel-related factors influence the bioburden in the air, including the number of people and human activities, such as the apparel worn by personnel and the frequency of the door of an OR opening [6, 15–18]. Most previous studies compared collection methods for microbial air [2, 10, 11, 16, 19]. There are few studies of the number of microorganisms in the air of an operating room and the factors of a working OR that influence this number. Identification of the factors that influence the number of bacteria is important for the development and implementation of effective preventive measures. Thus, our research hypothesis was that the microbial colony counts will be relatively lower in working ORs under a well-controlled ventilation system. Furthermore, due to the lack of more powerful documentary evidence, we wanted to demonstrate the important factors and their degree affecting the number of microbial colonies. The target population chosen included various types of 28 ORs. The main objectives of this study were to use active sampling methods to assess the outcomes on microbial colony counts in working ORs and to determine the outcomes on the factors influencing air contamination in a tertiary referral medical center**.**

## Methods

### Hospital and operating room environment

This prospective observation study was conducted at the 28 ORs of a medical center in Taiwan. More than 31,500 surgical procedures were performed in the ORs annually, with an average of approximately 100 surgical procedures per workday. Surgical procedures included neurosurgery, general surgery, colorectal surgery, orthopedic surgery, pediatric surgery, thoracic surgery, plastic surgery, urology surgery and transplant surgery. The types of surgical procedures were selected every workday for a purposive sampling technique. The air sampling conditions operated from Monday to Friday, May through August 2015. Patients who underwent surgery on holidays were excluded. The numbers of air sampling for the types of surgical procedure were selected according to its proportion in 2014, and the ORs were randomly selected. The day of air sampling, we checked surgical schedules including surgical procedures and surgery room in advance.

Each OR was equipped with a ventilation system with a high efficiency particulate air (HEPA) filter. The HEPA filters remove particles ≥0.3 μm in diameter with an efficiency of 99.97%. The ventilation systems of the ORs produced a minimum of 40 air changes of filtered air per hour (ACH) and 100% fresh air. The vertical laminar airflow systems were used to move particle-free air over the aseptic operating field at a uniform velocity (0.3~0.5 μm/s). Air conditioning equipment was also maintained and filter units replaced on a regular basis.

Indoor air temperature was kept at 19~23 °C and relative humidity (RH) at 30~70%. Equipment and environmental surfaces were routinely cleaned and decontaminated, and visible soiling was also added for processing. The doors of the ORs were always kept closed during surgical procedures except as needed for passage of equipment, personnel and patients.

### Sampling and data collection

A trained investigator with OR experience explained the purpose of the study and the methods to personnel before the study. The surgical team must follow their perioperative job specifications, and surgical aseptic techniques should be strictly enforced [17]. Each OR contained an Electrosurgery Unit (ESU) cart that was used in almost every procedure. This was selected as the ideal location for the air sampler because it was close to the surgical area and the surgical team. Therefore, the air sampler was set on the ESU cart, 1 m away from the ground and within 1 m of the operating table. These surgical procedures were collected from different ORs. We measured the microbiologic air counts in working ORs. The surgical stages at air microbial sampling were included before initial incision, during incision to wound closure, and after wound closure.

Microbiological air counts were measured using a one-stage viable impactor air sampler MAS-100 NT (Andersen sampler, Merck Inc., USA) at a flow rate of 100 L/min for 10 min (1000 L). The impactor was disinfected before and after each sampling. The collected air samples were plated onto trypticase soy agar and then incubated for 48 h at 35 °C. Colonies on the dishes were Gram stained, and the positive or negative bacteria were observed under a microscope at 1000X. Bacterial counts were expressed as colony forming units per cubic meter (cfu/m^3^). Bacterial genera were also identified. If fungi were isolated, further culture with selective medium would also be carried out. In order to reduce the detection bias, the laboratory personnel was blinded from the information of ORs and surgical procedures.

In addition to the collection of airborne microbes, data on potential influencing factors were collected, such as procedure-related operative characteristics and surgical environment (environmental- and personnel-related factors) characteristics (see Tables 1 and 2).

**Table 1:** Influence of procedure-related operative characteristics on microbial colony counts assessed by active sampling in working operating rooms

Variables	Number of samples *n* = 250 (%)	Mean bacterial count (cfu/m^3^)*	*p*-value
Surgery time# (mean ± SD)		2:53 ± 2:31	0.519
Surgery schedules			0.143
regular surgery	220 (88.0)	76.4 ± 45.8	
urgent surgery	30 (12.0)	89.7 ± 53.1	
Types of procedure			0.012
general surgery	104(41.6)	67.0 ± 44.7	
urology surgery	32(12.8)	89.5 ± 43.9	
colorectal surgery	24(9.6)	69.6 ± 37.5	
neurosurgery	23(9.2)	80.0 ± 40.2	
plastic surgery	20(8.0)	82.5 ± 55.7	
orthopedics surgery	19(7.6)	92.5 ± 55.2	
thoracic surgery	16(6.4)	84.1 ± 52.9	
pediatric surgery	9(3.6)	115.0 ± 30.3	
transplant surgery	3 (1.2)	123.0 ± 59.8	
Site of surgery			0.024
superficial incision	97(38.8)	68.3 ± 46.4	
deep incision	54(21.6)	88.3 ± 47.0	
organ/space	99(39.6)	81.8 ± 45.8	
Wound classification			0.061
clean	132(52.8)	72.1 ± 45.7	
clean-contaminated	65(26.0)	88.3 ± 47.5	
contaminated	35(14.0)	73.3 ± 39.9	
dirty	18(7.2)	92.8 ± 58.0	

*cfu colony-forming units; #Surgery time = during incision to wound closure

**Table 2:** Influence of surgical environment characteristics on microbial colony counts assessed by active sampling in working operating rooms

Variables	Number of samples *n* = 250 (%)	Mean bacterial count (cfu/m^3^)*	*p*-value
Indoor air temperature (°C), mean ± SD		19.2 ± 0.9	0.101
Indoor air relative humidity (%), mean ± SD		59.1 ± 4.4	0.998
Number of indoor staff, n		6.2 ± 1.8	0.001
2 persons	6 (2.4)	43.0 ± 46.3	
3 persons	16 (6.4)	57.6 ± 31.4	
4 persons	16 (6.4)	78.1 ± 41.1	
5 persons	33 (13.2)	75.0 ± 41.2	
6 persons	73 (29.2)	73.2 ± 43.7	
7 persons	62 (24.8)	76.4 ± 47.1	
8 persons	32 (12.8)	101.7 ± 52.9	
9 persons	6 (2.4)	109.7 ± 55.5	
10 persons and above	6 (2.4)	95–129	
Frequency of opening the operating room door during a surgical procedure			0.231
< 5	133 (53.2)	73.6 ± 46.8	
6–10	76 (30.4)	80.7 ± 45.1	
> 10	41 (16.4)	87.1 ± 49.4	
Sequence of the operation during the day			0.690
First	37 (14.8)	69.4 ± 47.0	
Second	56 (22.4)	73.4 ± 43.8	
Third	71 (28.4)	84.1 ± 50.9	
Fourth	46 (18.4)	83.0 ± 49.1	
Fifth	28 (11.2)	73.8 ± 41.6	
Sixth and above	12 (4.8)	74–124	
Surgical stage at air microbial sampling			<0.001
before initial incision	67 (26.8)	99.9 ± 55.7	
during incision to wound closure	170 (68.0)	66.9 ± 38.3	
after wound closure	13 (5.2)	110 ± 46.6	
The time of sampling			0.608
8:00–12:00	14 (5.6)	66.6 ± 42.3	
12:01–16:00	116 (46.4)	77.6 ± 48.9	
16:01–20:00	120 (48.0)	79.7 ± 48.9	
Total air microbiologic sampling microbial colony counts		78.0 ± 46.8	

*cfu colony-forming units

### Statistical analysis

Using t –test for a 2-sided = 0.05 at 80% power and 20% effect size by G^*^Power soft ware, the sample size of one group was determined to be 199. To account for up to 25% of potential samples lost, a total sample size of 250 surgical procedures was used. Data were expressed as the mean and standard deviation. Student’s t test, analysis of variance and simple Pearson correlation analyses for continuous data, and the chi-square or Fisher’s exact test for categorical data were used when appropriate. With microbial colonies as a dependent variable, multiple linear regression was performed to evaluate the significance of factors affecting airborne bacterial concentrations. Models were constructed using a purposeful selection of variables. We also checked for co-linearity between covariates. All tests were two-tailed; *p* values of less than 0.05 were considered statistically significant, and 95% confidence intervals (CIs) were calculated.

## Results

### Procedure-related operative characteristics

Table 1 lists the procedure-related operative characteristics and microbial colony counts. The study sample consisted of 250 surgical procedures, most which were regular surgeries (88%). The most frequent surgeries in the ORs were general surgeries (41.6%), urology surgeries (12.8%) and colorectal surgeries (9.6%). The most common sites of surgery were the organ/space site (39.6%), and the most common wound classification was clean wound (52.8%).

With air microbial colonies of the ORs as the dependent variable, there were significantly different variables (*p* < 0.05) by univariate analysis, including surgical procedures (*p* = 0.012) and site of procedure (*p* = 0.024). Of the surgical procedures, the mean number of colonies was the highest for transplant surgery at 123 ± 60 cfu/m^3^, and the lowest for general surgery at 67 ± 45 cfu/m^3^. The average number of microbial colonies was the highest in deep surgical sites (88.3 cfu/m^3^) and organ / space sites (88.3 cfu/m^3^), and the lowest in superficial sites (68.3 cfu/m^3^).

### Surgical environment characteristics

As shown in Table 2, the mean indoor temperature was 19.2 ± 0.9 °C and the mean indoor RH was 59.1 ± 4.4%; the mean number of indoor staff was 6 ± 2 persons, and in half of the cases (*n* = 133, 53.2%) the number of OR door openings during the surgical procedures was less than 5. The third surgery (*n* = 71, 28.4%) and the second surgery (*n* = 56, 22.4%) of the surgical sequence during one day were the most commonly sampled for bacterial colonies; the most frequent sampling period was from the first incision to wound closure (*n* = 170, 68%), and the most frequent time of sampling between 16:01–20:00 (*n* = 120, 48.0%). The results of univariate analysis found variables significantly affecting bacteria counts including the number of indoor staff (*p* = 0.001). According to air microbial sampling at the staging before the surgical procedure, the mean bacterial counts before the initial incision and after wound closure were significantly higher (43 cfu/m^3^, *p* = 0.001) than during incision to wound closure.

The overall mean number of bacterial colonies in the ORs was 78 ± 47 cfu/m^3^. Among these 250 samples, a total of 25 (10%) contained pathogens isolated from air in ORs; *Coagulase-negative staphylococcus* (n = 12, 4.8%) was the most common pathogen, followed by *Micrococcus spp.*, (*n* = 11, 4.4%) and *Staphylococcus* (*n* = 2, 0.8%). No fungi were cultured from the air samples.

### Factors influencing microbial colony counts

After controlling for potentially confounding factors for bacterial counts in ORs using a multiple linear analysis, we found statistically significant positive correlations (*r* = 0.10–0.35, *p* < 0.05) with bacterial counts for the type of surgery, site of procedure, number of indoor staff, frequency of OR door opening and surgical staging; the stage of surgical procedure had the highest correlation with the amount of bacteria (coefficient of determination, R^2^ = 12%).

We also performed a multiple linear regression model where the independent factors influencing bacterial colony counts included types of surgery: pediatric surgery was higher than adult surgery 29.85 cfu/m^3^ (95% CI 1.28–58.42, *p* = 0.041), site of procedure: deep incision and organ/space higher than superficial incision 20.19 cfu/m^3^ (95% CI 8.24–32.14, *p* = 0.001), number of indoor staff: each additional person increased indoor the bacterial colony counts by 4.93 cfu/m^3^ (95% CI 1.47–8.38, *p* = 0.005), surgical staging: before incision and after wound closure higher than during incision to wound closure 36.5 cfu/m^3^ (95% CI 24.76–48.25, *p* < 0.001), and indoor air temperature: each additional 1 °C increased indoor bacterial colony counts by 9.4 cfu/m^3^ (95% CI 1.61–17.18, *p* = 0.018) (Table 3).

**Table 3:** The factors affecting bacterial counts in operating rooms using linear regression analysis

Variables	Pearson Correlation	Pearson Correlation *p* -value	Regression Coefficients	Regression Coefficients Standard Error	Regression Coefficients 95% CI*	*R*egression *p*-value
Surgery time# (hour)	−.005	.470	−1.72	2.30	−6.25-2.80	.453
Surgery schedules (urgent surgery/regular surgery)	.093	.071	3.66	8.57	−13.21-20.54	.669
Type of surgery (pediatric surgery / adult surgery)	.153	.008	29.85	14.50	1.28–58.42	.041
Site of procedure (deep incision and organ/space / superficial incision)	.164	.005	20.19	6.07	8.24–32.14	.001
Wound classification (contaminated and dirty/ clean and clean-contaminated)	.021	.368	4.82	6.62	−8.23-17.87	.467
Indoor air temperature (°C)	.104	.051	9.40	3.95	1.61–17.18	.018
Indoor air relative humidity (%)	.001	.499	1.32	.81	−0.27-2.91	.104
Number of indoor staff (n)	.217	<.001	4.93	1.75	1.47–8.38	.005
Frequency of operating room door opening during a surgical procedure (n)	.109	.043	7.28	3.77	−0.15-14.70	.055
Sequence of the operation during the day	.065	.154	2.21	2.13	−2.00-6.41	.302
Time of sampling	.052	.205	3.65	4.45	−5.13-12.42	.413
Surgical staging (before incision and after wound closure /during incision to wound closure)	.346	<.001	36.50	5.96	24.76–48.25	<.001

*CI Confidence Interval; #Surgery time = during incision to wound closure

## Discussion

### Sampling and culture of microorganisms

The microbial concentration in an indoor OR is one of the extrinsically influencing factors for SSI. Despite our results demonstrated that free fungi and the number of mean bacterial colonies were far from the suggested levels during surgical activity despite a well-controlled OR ventilation system with a HEPA filter.

Inadequately filtered incoming air was the major source of fungal contamination; the suggested alert values were 4 cfu/m^3^ [20]. Most reports suggest that an acceptable bacterial limit for a working OR is below 180 cfu/m^3^ [6, 10, 16, 21]. However, differences in institutions, OR settings, sampling methods, study periods and sampling locations might account for some differences in microbial concentrations between studies. Pasquarella et al. performed microbial monitoring in 29 conventionally ventilated ORs over a three-year period; they found that bacterial contamination values in working ORs varied widely. The mean bacterial contamination was from 122 to 149.7 cfu/m^3^; however, 6%–27% of the samples had more than 180 cfu/m^3^ and maximum values of 798 cfu/m^3^ [10]. Another report found airborne bacterial counts ranging from 87 to 585 cfu/m^3^ [11]. In our study, the mean colony counts obtained in ORs were 78 ± 47 cfu/m^3^ (with the lowest counts in general surgery of 67 cfu/m^3^ to the highest in transplant surgery of 123 cfu/m^3^), which was much lower than 180 cfu/m^3^. The data of active sampling are known to be highly variable, and its possible influencing factors include the absorption of the medium, the amount of air drawn, and the size-selection sampling head designed [22]. We used the one-stage Andersen sampler to collect data on microbiological counts, unlike the sampling collection instruments used in previous study [10, 11]. In the recommendations of the guideline for prevention of SSI, conventional OR ventilation systems produce a minimum of about 15 ACH, three (20%) of which must be fresh air [5]. Several studies reported that air conditioning frequencies were set at 15 ACH in their ORs [10, 15, 16]. In comparison, the ORs of this study were taken with a higher standard air conditioning setting (40 ACH and 100% fresh air), which may have caused microbial colony counts lower than some of the other reports.

Our data also found that 4.4% (*n* = 11 of 250) of the samples had colony counts over 180 cfu/m^3^; of them, 82% (*n* = 9 of 11) of samples were taken before incision staging. Personnel movements were more frequent during the patients’ preoperative preparation period, such as laying surgical drapes. These behaviors may lead to an increase in the number of bacteria in the air.

Several reports noted that gram-positive bacteria, including *Staphylococcus* spp., *Bacillus* spp., and *Micrococcus* spp., often existed in the OR area, followed by gram-negative bacteria, such as *Acinetobacter* spp., *Moraxella* spp., *Pseudomonas* spp., and *Stenotrophomonas* spp. [15, 19, 23]. Of these bacteria, *S. aureus* was highest in all locations in the ORs and showed resistance to methicillin and ampicillin [5, 23, 24]. Because a disrupted skin barrier promotes skin colonization by microbes, these investigations detected pathogenic bacteria, such as *Staphylococcus* spp., *Acinetobacter* spp., and *Pseudomonas* spp., which are implicated in healthcare-associated infections in ORs [25]. These bacteria also cause outbreaks of SSIs [5]. Similar to previous reports, our data sampled from the ORs most frequently contained isolates of *staphylococci* (5.6%). In recent years, concern has been expressed about the risks posed by contaminated mechanical ventilation ductwork in hospital buildings. The role of environmental contamination in the spread of Gram-negative bacterial infections is being increasingly recognized [20].

### Factors influencing microbial colonies

The number of microbial colonies in the OR was influenced by a variety of causes; the main factors could be divided into indoor environmental- and personnel-related factors [1, 6, 15, 23]. For intraoperative characteristics, we found that the surgical procedure had a statistically significant effect (*p* = 0.012) on microbial colony counts. Of these procedures, the highest mean numbers of colonies were found during transplant surgery and pediatric surgery. The reasons maybe that transplant surgeries require a relatively high number of staff to participate in the operation, while more people enter into the OR during pediatric surgeries, such as interns and parents who accompany the child before anesthesia. Our results are similar to a previous report, that the mean colony counts during pediatrics surgery were the second highest of all surgeries [10]. We also used multiple regression to further control for covariance; the mean bacterial colony counts in pediatric surgery were significantly higher than in adult surgery (*p* = 0.041).

In addition, the site of procedure had a significantly positive correlation (*r* = 0.164, *p* = 0.005) with bacterial counts in the ORs; deep incision sites and organ/space sites had higher counts than superficial incision sites with mean bacterial counts of 20.19 cfu/m^3^ (p = 0.041). This may be because deep incision site and organ/space site surgery requires sewing subcutaneous tissue, fascia and muscle layers, so these operations are more difficult and complex, requiring more assistant physicians.

Several reports noted the number of staff and their activities also influence microorganism concentrations in the air of ORs [1, 15–17, 23]. The microbial level in OR air is directly proportional to the number of people moving about in the room [5, 18]. A study evaluated long-term variation in air quality in ORs. Their results indicated that the number of people and bacterial concentration were positively correlated (*r* = 0.36, *p* < 0.01). However, no significant correlation existed between the number of people in a space and the airborne bacterial concentration after adjusting for temperature, RH, and sampling location [23]. In the present study, the mean number of indoor staff was six people; the results also showed a significantly positive correlation (*r* = 0.217, *p* < 0.001) between the number of indoor staff and bacterial concentration. Even after adjusting for other potential confounders by multiple regression analysis, these two variables were still significantly correlated, with an increase in indoor colony counts with the addition of each person. Thus, the number of people entering the OR should be limited to necessary personnel [5, 7]. Hospitals should consider controlling the number of occupants (estimated maximum occupancy of 20 persons/1000 square feet) and increasing outdoor air requirements (15 cubic feet per minute/person) in OR areas to achieve acceptable indoor air quality. Further suggestions include using occupancy sensors for selected occupancy types for ventilation control [9].

Moreover, inferences can be derived from the three reports on the impact of personnel on the number of microbe colonies. Edmiston et al. recovered microorganisms in an empty OR and during vascular surgery using an impactor sampling device with a low microbial threshold; the total microbe concentration increased from fourfold to twelvefold in the presence of OR personnel [4]. Another report also revealed median bacterial counts of 80 cfu/m^3^ in working ORs, significantly (*p* < 0.001) higher than the values of 12 cfu/m^3^ in empty ORs [10]. Recently, Dai et al. conducted a study with microbiologic air sampling every 30 min from 7:00 AM-6 PM; the bacterial count results were the highest during preparation periods (during which the medical staff conducted preoperative procedures and postoperative cleaning) with a mean value of 377 cfu/m^3^ and were the lowest under static conditions (no medical activity), with a mean value of 162 cfu/m^3^ [11].

Our results were similar to previous studies demonstrating that the microbial colony counts had the greatest degree of correlation (*r* = 0.346, p < 0.001) with the surgical stage in the regression model; counts sampled before the incision stage and after the wound closure stage were higher than those during incision to wound closure stage with colony values of 36.5 cfu/m^3^; four-fifths of the microbial colonies detected over the threshold were also at the before incision stage. Our study also showed that the preparation period had the most frequent personnel activity, including door opening, covering and removing cloth sheets and other items, causing air fluctuations and raising dust in the process.

It may be these reasons that in the regression model after controlling for other variables, the affect of surgery time (during incision to wound closure) on bacterial counts in ORs was a negative regression coefficient. In addition, the ventilation systems are running continuously during this period. It had no statistically significant differences, and the 95% CI (−6.25–2.80) included positive regression coefficients.

Although turbulent ventilation was not related to wound contamination (*p* = 0.22), it was associated with an increased number of air microbial counts (*p* < 0.001) [26]. Air in the OR may contain microbial-laden dust, lint, skin squames, or respiratory droplets, so microorganisms can collect on OR environment surfaces [2, 15, 20, 24]. Thus, conventional cleaning is also necessary.

Opening the door of the OR was not only one of the causes of fluctuations in indoor air but also affects the OR air ventilation so that it cannot completely remove contaminants from the air [4]. We found a significant positive correlation (*r* = 0.109, *p* = 0.043) between the frequency of opening the door of the OR and bacterial counts. Doors should be kept closed in surgical procedures except as needed.

Additionally, the number of people in the OR was found to be significantly positively correlated with the air temperature [15]. Temperature and humidity influence viral, bacterial and fungal particles [3, 27]. Therefore, environmental factors substantially influence the efficacy of airborne disease transmission [3, 28]. The present study was similar to a previous report that found no significant correlation between RH and bacterial concentrations [15]. Although the indoor temperature was not significantly related to the number of microbial colonies, the colony count significantly increased by 9.4 cfu/m^3^ with each additional 1 °C (*p* = 0.018).

This study design could reflect the actual microbial concentrations during surgical procedures. Several potential limitations of our study should be noted. First, this study was not performed at multiple locations, only in a single medical center with 28 ORs for indoor microbial sampling and data collection, so inferences to other levels of hospitals should be drawn with caution. However, the advantage of this situation was that the settings and equipment of the each OR environment and the experience of the personnel were similar for all working ORs and surgical procedures; this also reduced the bias of the assessment-influencing factors. Second, we excluded bacterial air sampling and data collection on holidays. Final, our purposes of the study were to assess microbial colony counts in working ORs and to determine the factors influencing air contamination. Thus, only bacterial genera were identified. Our study did not detect the bacterial species and perform the molecular typing. We suggest that future studies can add molecular identification, especially in a study of the relationship between microbes in the air and SSIs.

## Conclusions

In the present study, the mean microbial colony counts obtained by active sampling in different working ORs were low. Independent risk factors that influence the number of microbial colonies in operation were the type of surgery, site of procedure, number of indoor staff, surgical staging, and indoor air temperature. We suggest that ORs doing complex surgeries with more surgical personnel present should increase the frequency of air exchanges. In addition to a well-controlled OR ventilation system, it is important to regulate the number of people present. Implementation of infection control and traffic control together will improve adherence.

## Abbreviations

ACH: Air changes of filtered air
Cfu: Colony forming units
CI: Confidence interval
ESU: Electrosurgery unit
HEPA: High efficiency particulate air
OR: Operating room
RH: Relative humidity
SSI: Surgical site infection
